# Neuroblastoma stem cells and CFC1

**DOI:** 10.18632/oncotarget.18491

**Published:** 2017-06-15

**Authors:** Shoma Tsubota, Kenji Kadomatsu

**Affiliations:** Department of Biochemistry, Nagoya University Graduate School of Medicine, Nagoya, Japan

**Keywords:** neuroblastoma, cancer stem cells, spheroid culture, CFC1, activin

Cancer stem cells (CSCs) have been implicated in many cancers and build up an intratumor heterogeneity bestowing multiple competences on the tumor for metastasis and resistance to anti-cancer drugs [1]. Neuroblastoma is a childhood cancer arising mostly in adrenal gland and sympathetic ganglia [2]. CSCs hypothesis in neuroblastoma recently appeared [3] and was experimentally supported by the ability of sphere formation in vitro and tumor formation in immunodeficient mice [4]. Accordingly, several methods of sphere culture have been established [4, 5]. Hansford LM et al. [4] and Takenobu H et al. [6] reported the spheroid culture from bone marrow of neuroblastoma patients.

The study by Chikaraishi K et al. [7] in this issue of Oncotarget utilized a spheroid culture method to identify molecules highly expressed in patient-derived spheres. The authors carried out transcriptomic analysis of spheres derived from bone marrow-metastasized tumors of two advanced neuroblastomas (MYCN amplified and stage 4). They identified 206 commonly upregulated genes in these spheres and focused on CFC1 which was not investigated in neuroblastoma so far. In addition, they found that the expression of activin receptor 2A (ACVR2A) was reversely correlated with prognosis although it was not changed upon sphere formation. Through unique approaches employing sphere formation of neuroblastoma cell lines, two lines of findings, i.e., CFC1 and the activin signaling, were linked together. Thus, knockdown of CFC1 by short-hairpin RNAs inhibited sphere formation of primary neuroblastoma cells and the neuroblastoma cell lines in vitro. In contrast, exogenous CFC1 expression in NGP and NB-39-nu neuroblastoma cell lines promoted cell proliferation, sphere formation, colony formation in soft agar and subcutaneous tumor growth. A microarray analysis for NGP neuroblastoma cells expressing CFC1 revealed that the upregulated genes are associated with “differentiation pathway” and three transforming growth factor-β (TGF-β) signaling pathways. Interestingly, exogenous expression of CFC1 in these cells attenuated the activin A-induced cellular and molecular differentiation phenotypes. Moreover, they observed sphere formation of NGP cells when the cells were treated with the activin receptor inhibitor sb431542 as well as overexpressed with CFC1, suggesting that the inhibition of activin signaling is required for sphere formation in neuroblastoma cells.

Cryptic protein, which is encoded by CFC1 gene (crypto, FRL-1, cryptic family 1), belongs to the EGF-CFC family, and is a membrane-bound co-receptor which regulates signaling of the TGF-β superfamily. In human, TGF-β superfamily consists of more than thirty ligands. Importantly, the ligands including BMPs, activins, NODAL and GDFs are known to control self-renewal ability and differentiation of CSCs. In this context, it is of note that the importance of the activin and CFC1 axis in CSCs in neuroblastoma was highlighted for the first time by Chikaraishi K et al. [7]. The upstream molecular mechanisms of CFC1 expression and the mechanisms underlying CFC1-meditated suppression of the activin signaling need to be investigated in future studies, and are of importance, in particular, to make the CFC1-activin axis a potential therapeutic target in neuroblastoma.

Neuroblastoma is thought to be a consequence of disorganized development of the sympathoadrenal lineage. Indeed, driver gene mutations are rare in neuroblastoma [8] and epigenetic regulation may play more important roles in tumorigenesis of neuroblastoma than in adult cancers. This may partly explain why success rate of sphere culture of primary neuroblastoma is low. Even though some markers including CD133 have been proposed [6], reliable, useful markers for neuroblastoma CSCs have not been established. The paper by Chikaraishi K et al. [7] added another candidate marker for them.
